# Comprehensive Review of Endometrial Cancer: New Molecular and FIGO Classification and Recent Treatment Changes

**DOI:** 10.3390/jcm14041385

**Published:** 2025-02-19

**Authors:** Maria-Bianca Anca-Stanciu, Andrei Manu, Maria Victoria Olinca, Cătălin Coroleucă, Diana-Elena Comandașu, Ciprian Andrei Coroleuca, Calina Maier, Elvira Bratila

**Affiliations:** 1 Department of Gynecology, Carol Davila University of Medicine and Pharmacy, 050474 Bucharest, Romania; bianca_stanciu16@yahoo.com (M.-B.A.-S.); andrei.manu@umfcd.ro (A.M.); diana.comandasu@yahoo.com (D.-E.C.); cip_coroleuca@yahoo.com (C.A.C.); calina.maier@umfcd.ro (C.M.); elvirabarbulea@gmail.com (E.B.); 2 Panait Sirbu Obstetrics and Gynaecology Hospital Bucharest, 060251 Bucharest, Romania; ccoroleuca@yahoo.com; 3 Department of Morphological Sciences, Carol Davila University of Medicine and Pharmacy, 050474 Bucharest, Romania

**Keywords:** endometrial cancer, FIGO classification, molecular classification, The Cancer Genome Atlas (TCGA), POLE ultramutated, microsatellite instability-high (MSI-H), copy-number low (CNL), copy-number high (CNH), immunotherapy, targeted therapies, personalized medicine

## Abstract

Endometrial cancer (EC) is the most common gynecologic malignancy in developed countries, with rising incidence due to aging populations and obesity-related factors. This review explores the evolving molecular and FIGO classifications of EC, highlighting their significance in diagnosis, prognosis, and personalized treatment strategies. Molecular subtyping based on The Cancer Genome Atlas (TCGA) classification offers a more precise understanding of EC, dividing it into POLE ultramutated, microsatellite instability-high (MSI-H), copy-number low (CNL), and copy-number high (CNH) subtypes. Each subgroup has distinct genetic, histological, and prognostic characteristics. Recent updates to the FIGO staging system incorporate molecular features, allowing for more tailored treatment approaches. Advances in immunotherapy, targeted therapies, and novel therapeutic combinations have reshaped clinical management. This review emphasizes the integration of molecular diagnostics into routine practice, outlining challenges and future perspectives in managing EC for improved patient outcomes.

## 1. Introduction

The incidence of endometrial cancer (EC) is gradually increasing. At present, the lifetime risk of developing EC in a woman is about 2.6–3% [1]. As a result of this, the increased prevalence of other obesity-related conditions, and the aging of the population, EC presents a considerable and growing health burden [2]. Fortunately, most ECs are diagnosed at an early stage due to the relatively early onset of symptoms, which usually reflect the localized disease [3]. The main presenting symptom is postmenopausal or irregular vaginal bleeding, which is reported in over 90% of patients. However, it is important to note that despite the generally good prognosis of most ECs, the mortality rate has not decreased in the past decades [4].

EC is the most common gynecologic cancer in developed countries. Through this updated review, the demographics, risk factors, molecular features, diagnostic evaluation, prognostic assessments, and treatment options are concisely addressed [5]. Most importantly, this review looks to provide the most current information regarding recent advances in novel molecular and genetic EC classifications, which have become increasingly significant [6]. The new FIGO staging system and treatment changes are also briefly reviewed [7].

### 1.1. Background and Significance

EC is the most common gynecologic cancer in developed countries. An increase in the incidence of EC is expected globally, related to the epidemic of obesity, metabolic syndrome, and mainly aging of the world population [6]. The traditional classification of EC by histology has provided little therapeutic guidance beyond prognosis. Recently, The Cancer Genome Atlas (TCGA) project and others have proposed molecular classification in an attempt to improve understanding of EC [8]. Stage, grade, histology, lymphovascular space invasion (LVSI), and hormone receptor expression have been proposed to be included in the EC Biomarkers Alliance (ECBA) group in addition to molecular classification [9]. The FIGO committee ratified a proposal for staging modifications to better stratify patients according to prognosis in the early stage by including LVSI and cervical stromal invasion. The aim of this review was to summarize the summary of ECBA group recommendations and the most recent updates in molecular characteristics, staging, and treatment changes according to the literature [10].

### 1.2. Objective of the Review

This paper has two main scopes. The first is the revision of the new molecular and the FIGO classifications of endometrial cancer [11]. The second is the discussion of recent treatment changes. In Section 3, we will discuss the new molecular classifications of EC. We will review the classification proposed by TCGA, the PORTEC molecular classification, the ESMO and the MoLINo proposals, and other classifications. We will also consider the advances and the ongoing challenges of the molecular classifications in clinical practice [12].

After a brief introduction, in Section 2, we will present an overview of the epidemiology of uterine cancer [13]. This will focus on the incidence and mortality, as well as the factors that influence the risk of developing endometrial cancer [14]. We will describe the protective and enhancing role of the factors that influence endometrial cancer [15]. We will also review the risk factors that are specifically associated with the development of the molecular EC subtypes [16].

The objective of this review is to examine the new trends in the molecular and the recent FIGO classification and treatment of EC [17]. Recently, EC has revealed new molecular classifications which help to identify the different genetic endometrial tumor types and to selectively target treatment at the molecular level [18]. This is designed to specifically inhibit the pathways that are activated or deregulated in each type [19]. It also realizes the potential treatment changes in EC with the use of the new classifications [20]. The review will also discuss the latest changes adopted by the FIGO staging and the treatment of EC [21].

## 2. Understanding EC

The next step will be the review of recent classification, preferably the latest up-to-date or just one step behind, changes in treatment, the translation and applicability of the new molecular and pathologic classification in clinical use or in routine practice, and finally, the future implementation in the clinical setting [22,23]. The new understanding at the molecular level in association with coexisting conditions, genetic abnormalities, immune checkpoint, cell cycle regulation, and specific affected pathways, and the collaboration of molecular and pathologic, the integration system of staging that takes into account radiologic, surgical, and pathologic findings should guide the future management of EC in a tailored, personalized, and specific manner to achieve the best outcomes with the least toxic therapy [17,24].

Understanding EC starts by digging into its etiology, the risks, and the factors that contribute to endometrial development. This sets the stage for discussions on prevention, early detection, and the unique biological characteristics of the disease [8,25,26]. In summary, a significant portion of EC cases are linked to conditions such as diabetes, obesity, unopposed estrogen exposure, and metabolic syndrome. These connections highlight the need for a deeper understanding of the distinct biology of EC, allowing for the development of more effective, targeted therapies that consider specific pathological variants, molecular risk classifications, and accompanying health conditions [8,20,27,28].

### 2.1. Epidemiology and Risk Factors

Known risk factors for EC include excess estrogen (unopposed estrogen exposure), late menopause, early menarche, nulliparity, infertility, polycystic ovary syndrome, hormone replacement therapy, tamoxifen, obesity, diabetes, and hypertension [7]. Protective factors include progestins, pregnancy, smoking, and physical activity. There are strong hormonal and metabolic components to EC etiology [9]. Up to 40% to 50% of EC cases may be related to excess estrogen. For this reason, EC is more strongly associated with elements of the metabolic syndrome and excess body weight than with other hormone-dependent cancers. Postmenopausal women with elevated estrogen levels, as observed in obese women, have a two- to twelvefold increased risk of EC [2]. The elevated risk persists even if the woman is taking estrogen-lowering aromatase inhibitors [9]. An estimated 20% to 57% of EC cases are related to excess body weight, and 14% could be prevented if women were of normal weight [29]. EC primarily affects postmenopausal women and has a median age at diagnosis of 63 to 66 years [30,31].

Worldwide, EC ranks seventh among all female cancers, with the majority of cases occurring between 65 and 75 years of age [30]. In Europe, EC ranks fourth among women with cancers, with an incidence of 12.9–20.2:100,000 and a low mortality rate of 2.0–2.7:10,000 [30,31]. It is the most common gynecologic cancer and the fourth most common cancer in women in the United States. The overall lifetime risk of developing EC in the United States is one in 36 (2.7%) [32]. In 2015, 54,870 new cases and 10,170 cancer-related deaths were estimated to occur in the United States. The incidence of EC is rising, primarily because of increasing numbers of aging, obese women [32]. Racial and ethnic disparities in EC incidence are observed; non-Hispanic white and black women have higher incidences than Hispanic, Asian, and American Indian/Alaska Native women [7]. Early detection of EC would significantly improve patient outcomes, as the survival rate for early-stage EC is over 90%, compared to less than 20% for advanced disease. Early diagnosis allows for less aggressive treatments and better quality of life, particularly for younger women who may benefit from uterus-sparing management options [33]. Transvaginal ultrasound (TVUS) and magnetic resonance imaging (MRI) are valuable tools in the preoperative assessment of endometrial cancer, offering high accuracy in evaluating myometrial invasion and cervical involvement. While TVUS is a cost-effective and widely accessible method, MRI provides superior soft-tissue contrast, making it particularly reliable for comprehensive staging and treatment planning [33]. Histopathological samples for diagnosing endometrial cancer can be obtained through several methods, including dilation and curettage (D&C), which is usually performed under general anesthesia, or via less invasive office-based procedures such as Pipelle aspiration biopsies. These office methods are effective and convenient for obtaining endometrial tissue with minimal discomfort. In selected cases, particularly those involving fertility-preserving management, hysteroscopy can be utilized to directly visualize the uterine cavity and obtain targeted biopsies, ensuring accurate diagnosis and tailored treatment planning [34].

### 2.2. Histopathological Classification Before Molecular Era

Over the last decade, a vast number of large-scale molecular studies have established a plethora of molecular alterations that have transformed and greatly improved our understanding of EC, unveiled the highly heterogeneous nature of these carcinomas, surpassed the many limitations posed by the histopathological classification, and have even spurred proposals to completely abolish the old classification [35]. Despite lacking in some respects, the molecular classification represents a groundbreaking advancement alongside histopathology, adding important additional information to direct future studies and novel treatment strategies, and to ultimately further our ability to accurately predict patient outcomes [35]. While further refinement is required, molecular classification is already becoming established in routine pathology [30]. This review aims to summarize the most important molecular features in each of the major molecularly defined groups of EC and highlights recent major changes in the approach [36].

EC is the most common gynecological malignancy in developed countries. Simplified, it is divided into type I endometrioid EC, representing most cases, and non-type I EC, which includes non-endometrioid EC [35]. Before the molecular era, the histopathological classification suffered from considerable inter-observer variability with poor reproducibility [37]. This led to the incorporation of the assessment of tumor cell growth patterns (gland forming, solid/partially solid, and non-squamous dedifferentiated) by the International Collaboration on Cancer Reporting (ICCR) and the Union for International Cancer Control (UICC) in their most recent published guidelines in the attempt to increase objectivity and reproducibility [38].

## 3. Molecular Classification of EC

**Figure 1:**
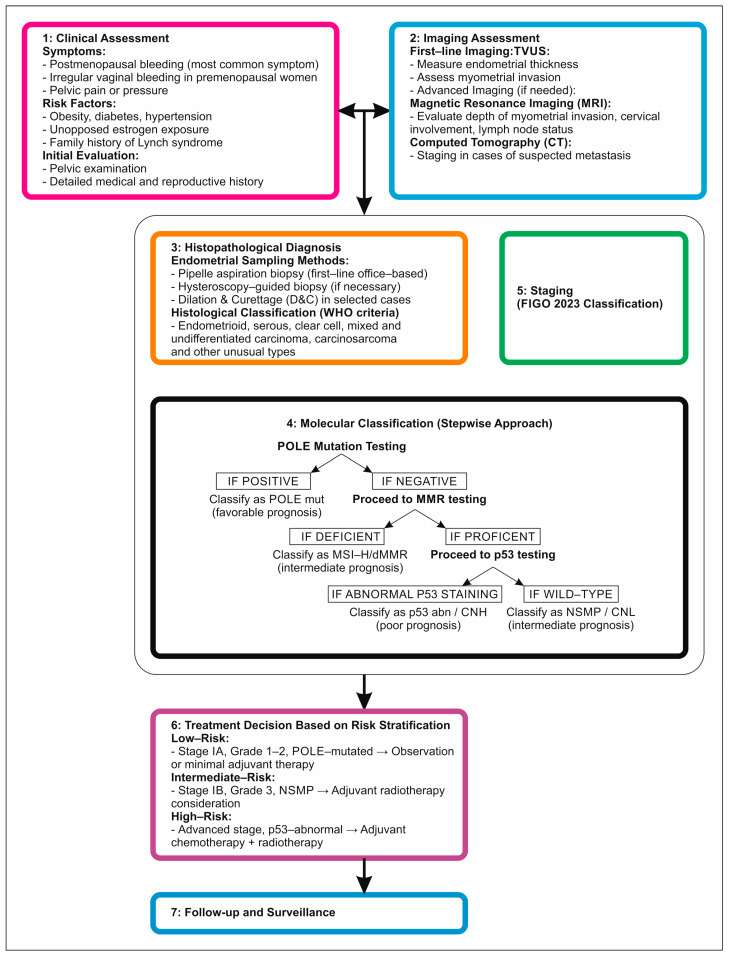
Algorithm of diagnostic in EC.

EC is traditionally divided into two types. Type I cancers are low-grade, estrogen-dependent lesions that develop in the setting of endometrial hyperplasia and generally have a good prognosis [39]. They are often found in the early stage and are effectively treated with surgery and hormonal therapy [40]. Type II cancers are high-grade, estrogen-independent tumors that arise from atrophic endometrium or a precursor lesion and are more likely to present at an advanced stage. Type II cancers have a worse prognosis than Type I cancers [41]. In contrast to type I EC, type II cancers have been poorly studied. They account for about 10–20% of cases, and the vast majority belong to the serous-like subgroup [41,42]. Although the dualistic model has provided a foundation for understanding EC’s pathogenesis and guiding treatment, the clinical and biological heterogeneity of these tumors is not completely captured [43]. This classification system, while useful, had limitations due to significant interobserver variability, especially in high-grade tumors, and did not always correlate well with clinical outcomes. Several recent advances in EC’s molecular features have led to the development of a novel classification that promises to better stratify patients based on prognosis and treatment response [44]. Molecular profiling studies have revealed that EC is far more heterogeneous than previously believed to be at least four distinct molecular subgroups. These molecular subgroups largely correspond to the traditional histology-based classification, implying different etiologies and pathways of carcinogenesis [45]. We have summarized the diagnosis and management of EC in a flow chart (Figure 1).

### 3.1. The Cancer Genome Atlas (TCGA) Project

The TCGA research network used multiple genomic platforms to identify integrated proteogenomic characterization of human colon and rectal cancer, correlative analysis of somatic and germline alterations, and cell line model of analyses of transposable element expression, among other novel approaches [46]. The inclusion of proteomic, phosphoproteomic, and other data types represents the evolution of the TCGA network as well as the larger cancer research community toward integrative and multi-dimensional analyses [12]. Since the endometrial carcinoma TCGA project provided a comprehensive new understanding of the disease using a similar integrative approach, we review these recent major discoveries. We also discuss other recent publications that describe key molecular, classification, and clinical evolution changes in the field of endometrial carcinoma, which are mainly enabled by TCGA project data and analyses.

The TCGA project has put forward comprehensive molecular characterizations of several cancer types using large-scale genome analysis. EC was, in fact, the very first gynecological cancer studied by the TCGA group [39]. The EC TCGA project contributed a dualistic model that divides EC into ultramutated and hypermutated types that are microsatellite instable and copy number low, and copy number high and microsatellite stable, respectively [46]. The TCGA team also described the most frequently mutated genes in EC, including PTEN, CTNNB1, PIK3CA, and KRAS, as well as the mutation and somatic copy number alteration burden of the other histological types of the disease, e.g., serous uterine carcinoma [41,47].

### 3.2. Key Molecular Subtypes

The classification of endometrial cancer in the molecular era has been significantly advanced by the TCGA project, which stratified endometrial carcinomas into four distinct molecular subgroups. The ESGO/ESTRO/ESP guidelines integrate the TCGA molecular classification into clinical practice by using more affordable methods while still providing a framework for risk stratification and management of endometrial carcinoma POLE-mutated, mismatch repair (MMR)-deficient, p53-abnormal, and “no specific molecular profile” (NSMP). This classification is achieved through immunohistochemistry (IHC) for markers like p53 and mismatch repair proteins (MLH1, PMS2, MSH2, and MSH6) and targeted sequencing of the POLE exonuclease domain [40] and has been shown to outperform traditional histologic classification in predicting outcomes and guiding therapy, including the use of targeted therapies and immunotherapy. These subgroups are resumed in Table 1 and discussed in detail below.

POLE ultramutated EC/POLE-mutated is a subtype of EC characterized by mutations in the exonuclease domain of the DNA polymerase epsilon (POLE) gene. These mutations lead to an ultrahigh somatic mutation rate, which is one of the highest among human tumors [42]. According to the TCGA project, POLE ultramutated ECs account for approximately 7–12% of endometrial cancers and are predominantly of endometrioid histology [43]. These tumors are often high-grade and exhibit significant lymphocytic infiltration, which is indicative of a strong immune response [44]. Despite their high-grade appearance, these tumors are associated with a favorable prognosis. Patients with POLE ultramutated tumors generally have excellent outcomes, with significantly lower rates of recurrence and higher overall survival compared to other molecular subtypes of endometrial cancer [44]. The favorable prognosis is thought to be due to the high mutational burden, which leads to the production of numerous neoantigens that elicit a strong antitumor immune response. Recent studies show increased infiltration of cytotoxic T-cells and upregulation of immune response genes in POLE-mutant tumors [40].

Microsatellite instability-high (MSI-H) endometrial carcinoma/mismatch repair deficient (dMMR) is characterized by a high frequency of mutations due to defects in the mismatch repair (MMR) system. This defect leads to the accumulation of errors during DNA replication, resulting in a hypermutated phenotype. dMMRd endometrial carcinomas account for approximately 30% of endometrial cancers and are associated with an intermediate prognosis [48]. The MMR system typically involves proteins such as MLH1, MSH2, MSH6, and PMS2. Deficiencies in these proteins can be detected through immunohistochemistry, which has become the standard diagnostic method for identifying dMMR tumors. The most common cause of MMR deficiency in sporadic ECs is MLH1 promoter hypermethylation, which silences the MLH1 gene. Clinically, dMMR endometrial cancers often present with distinct features. Patients with MLH1 promoter hypermethylation tend to be older, more obese, and have more advanced diseases at diagnosis compared to those with germline or somatic MMR mutations. dMMR tumors are also characterized by increased immune cell infiltration, including activated cytotoxic T lymphocytes and PD-L1 expressing cells, which may have implications for immunotherapy. The presence of dMMR in EC has significant therapeutic implications. dMMR tumors are more likely to respond to immune checkpoint inhibitors, such as pembrolizumab, which has been approved for the treatment of dMMR solid tumors that have progressed following prior treatment [49].

Copy-number low (CNL) endometrial carcinoma/“no specific molecular profile” (NSMP) is a molecular subtype of endometrial cancer characterized by microsatellite stability, and an intermediate prognosis. NSMP is identified by the absence of significant copy number alterations and lacks the high mutation burden seen in POLE mutated tumors or the extensive copy number changes seen in p53 abnormal tumors. The prognosis for patients with endometrial carcinoma is generally intermediate, falling between the favorable prognosis of POLE ultramutated tumors and the poor prognosis of CNH tumors [50]. CNL endometrial carcinomas are typically associated with mutations in genes such as PTEN, PIK3CA, and CTNNB1.

CTNNB1 mutations, particularly those located in exon 3 of the CTNNB1 gene encoding β-catenin, have emerged as significant factors influencing the clinicopathologic features and prognostic outcomes in endometrial carcinoma. These mutations are strongly associated with the activation of the Wnt/β-catenin signaling pathway, a key driver of tumorigenesis. Despite being more common in low-grade, early-stage tumors, CTNNB1-mutated tumors exhibit a paradoxically higher risk of recurrence compared to their wild-type counterparts. A meta-analysis has demonstrated that CTNNB1 mutations significantly increase the odds of tumor recurrence and correlate with worse disease-free survival among patients lacking a specific molecular profile (NSMP) [51]. Immunohistochemical analysis for nuclear β-catenin expression is considered a reliable surrogate marker for detecting CTNNB1 mutations, with high sensitivity and specificity [52], proving to be useful in clinical practice to identify patients who may benefit from intensified surveillance or adjuvant therapy. Preclinical studies have shown that inhibitors like PRI-724 and SM04690 target the Wnt/β-catenin signaling pathway [53] and anti-angiogenesis agents like Bevacizumab through their effects on tumor vasculature [13], have demonstrated preferential efficacy in CTNNB1-mutated tumors, potentially enhancing treatment outcomes.

The NSMP tumors often exhibit diverse histopathological features, including squamous differentiation and mucinous differentiation, which are correlated with specific genetic alterations [54]. The heterogeneity within the CNL group suggests that further stratification based on additional clinicopathological characteristics and molecular markers may be beneficial for more precise prognostication and personalized treatment approaches [55].

Copy-number high (CNH) endometrial carcinoma/p53 abnormal is a molecular subtype of endometrial cancer characterized by frequent copy-number alterations, a low mutational burden, and near-universal TP53 mutations. This subtype is associated with high-grade endometrioid and serous carcinomas and is known for its poor prognosis [56].

The CNH subtype is identified by the TCGA and is marked by abnormal p53 expression due to TP53 mutations. These tumors often present at advanced stages and are associated with aggressive clinical behavior, including higher rates of recurrence and reduced disease-free survival. The NCCN guidelines highlight that p53 abnormal ECs require multimodality treatment, which almost always includes chemotherapy, due to their aggressive nature [57].

**Table 1:** Molecular subtypes of EC—general characteristics [57].

Subtype (TGGA and ESGO/ESTRO/ESP)	Characteristics
POLE ultramutated/POLE—mut	mutations in the DNA polymerase epsilon (POLE) exonuclease domain generally, have a favorable prognosis
MSI-H/dMMR	exhibit a high frequency of mutations due to defects in the mismatch repair system have an intermediate prognosis
CNL/NSMP	typically, microsatellite stable have an intermediate prognosis
CNH/P53 abn	often associated with TP53 mutations and abnormal p53 expression generally, have the poorest prognosis

However, resource limitations necessitate prioritization for cases where molecular data directly influence management, such as high-grade tumors domain. Standardized algorithms and surrogate markers have streamlined this process, making molecular diagnostics feasible in routine clinical practice domain [40].

1. Step 1: POLE Mutation Analysis
  - Perform POLE exonuclease domain mutation analysis.
  - If positive, classify it as POLEmut and no further molecular testing is needed.
2. Step 2: Mismatch Repair (MMR) Testing.
  - If POLE is negative, test for MMR deficiency using immunohistochemistry (IHC) for proteins MLH1, PMS2, MSH2, and MSH6.
  - If MMR deficiency (loss of one or more proteins) is detected, classify as dMMR.
3. Step 3: P53 Immunohistochemistry Testing
  - If MMR testing is normal (proficient), perform P53 IHC testing.
  - If P53 shows an abnormal/mutant-like pattern, classify it as P53-abn.
4. Step 4: Final Classification
  - If POLE, MMR, and P53 are all negative or normal, classify them as NSMP.

The present guidelines increasingly recognize the importance of incorporating molecular features (particularly MMR, POLE, and p53) into the standard diagnostic workflow for endometrial cancer. However, the extent and timing of recommended molecular testing vary among organizations [58] (Table 2):

1. WHO Classification (2020)
  ○ Recommends integration of molecular analysis (MMR, POLE, and p53) in the pathologic evaluation of endometrial cancers, especially high-grade or ambiguous histology.
  ○ Promotes universal MMR deficiency testing or, at minimum, MMR testing in selected cases to identify hereditary predisposition (Lynch syndrome).
2. ESGO/ESTRO/ESP Guidelines (2021)
  ○ Strongly endorse universal MMR IHC testing in EC.
  ○ Suggest additional testing for POLE mutations in high-grade or early-stage, high-intermediate-risk tumors to guide adjuvant therapy decisions.
  ○ Recommend p53 IHC to distinguish between p53-wild-type vs. p53-abnormal tumors, aiding risk stratification.
3. ESMO (European Society of Medical Oncology)
  ○ Aligns with recommendations for universal or near-universal testing for MMR deficiency.
  ○ Acknowledges the significance of POLE status in prognostication.
  ○ Emphasizes integrated histopathological and molecular classification to personalize treatment decisions.
4. NCCN (National Comprehensive Cancer Network)
  ○ Advises universal screening for Lynch syndrome (by MMR IHC or MSI testing) in newly diagnosed EC.
  ○ Recommends consideration of p53 and/or POLE mutation testing in selected cases to refine prognostic risk categories.
5. SGO (Society of Gynecologic Oncology)
  ○ Recommends universal MMR deficiency testing for Lynch syndrome screening.
  ○ Encourages the use of molecular classification (MMR, p53, and POLE) in research/academic settings and increasingly in clinical practice for risk stratification, though the degree of routine adoption may vary.

The identification of these four main molecular subgroups has not only provided us with a better understanding of the disease but may also serve as an aid in guiding treatment decisions [12]. For example, patients with serous-like tumors generally do not benefit from adjuvant radiotherapy, unlike the other subgroups [57]. Some authors, in fact, propose that molecular subtyping should replace the current histological classification in order to guide treatment decisions [19]. Indications for performing lymphadenectomy might also be based on the molecular subgroup, given that the risk of lymph node metastasis varies significantly between the subgroups [59]. In the future, specific targeted therapies, which are currently being explored for some of the molecular subgroups, may further increase the importance of molecular classification [60].

### 3.3. Clinical Implications

EC is considered to be a hormone-dependent cancer, and approximately 80% of patients present with symptoms related to postmenopausal bleeding and are diagnosed at an early stage [61]. Some patients are at a higher risk for EC due to hereditary nonpolyposis colorectal cancer (HNPCC), which is associated with the mismatch repair genes. These patients have an increased risk for EC at a relatively young age that is independent of hormone exposure [61].

The European Society of Gynaecological Oncology (ESGO) 2021 guidelines provide detailed recommendations for the treatment of endometrial cancer based on the stage of the disease [62].

Stage I: For patients with Stage I disease, treatment primarily involves a total hysterectomy with bilateral salpingo-oophorectomy. Lymph node assessment is performed based on specific risk factors such as tumor grade and depth of myometrial invasion. Minimally invasive surgery is the preferred approach when feasible [63].

Stage II: In Stage II disease, where the cancer invades the cervical stroma but remains confined to the uterus, a radical hysterectomy with bilateral salpingo-oophorectomy and pelvic lymph node dissection is recommended. Adjuvant radiotherapy may be added depending on pathological findings and individual risk assessments [63].

Stage III: For Stage III disease, characterized by local and regional spread, a combination of surgery, chemotherapy, and radiotherapy is used. The extent of surgical resection depends on the tumor’s spread, and adjuvant therapies are tailored to the patient’s risk profile [63].

Stage IV: In Stage IV disease, where the cancer has metastasized to distant organs, systemic therapy with chemotherapy is the cornerstone of treatment. Radiotherapy or surgery may be employed for symptom management and local control, with a personalized approach considering the patient’s overall health and preferences [63].

These guidelines emphasize the importance of a multidisciplinary approach, considering tumor histology, molecular characteristics, and patient comorbidities to optimize treatment outcomes [63].

Early-stage patients are generally considered to be at low risk for recurrence, and only observation after surgery is recommended without additional treatment [64]. However, some patients who were staged as IB according to the old FIGO criteria had type II disease and had a poorer outcome. Adjuvant therapy was generally offered to patients with advanced-stage disease, but the treatment did not affect 5-year survival [16]. Patients diagnosed as having stage IIIA disease, according to the old FIGO staging, were very rare, and these changes are not expected to have a major impact on clinical practice [26]. There are significant differences in patients with stage IVA and IVB disease, and the new classification emphasizes this by upstaging these patients [3].

In addition to the correlation between molecular subtypes and prognosis, the integration of molecular markers into staging has led to significant stage migration. For instance, patients with p53abn tumors are often upstaged due to their poor prognosis, while those with POLEmut tumors may be downstaged due to their favorable outcomes. This reclassification impacts treatment decisions, such as the need for adjuvant therapy [64]. Molecular classification informs treatment decisions beyond traditional histopathological assessment. Patients with POLEmut tumors might avoid unnecessary aggressive treatments, while those with p53abn tumors might receive more intensive therapy. MMRd tumors may benefit from immunotherapy, such as pembrolizumab, particularly in recurrent settings [61].

## 4. FIGO Classification Updates

The FIGO 2023 system, incorporating molecular classification, has shown superior prognostic accuracy compared to the 2009 system. Studies have demonstrated that the 2023 system better predicts disease-specific survival and progression-free survival, particularly in early-stage disease [63].

The 2023 system introduces new substages within Stage I (IA1, IA2, IA3, IB, and IC) and Stage II (IIA, IIB, and IIC) based on histological type, depth of myometrial invasion, and lymphovascular space invasion (LVSI). It allows histological and anatomical refinements by differentiating between non-aggressive and aggressive histological types and includes specific criteria for LVSI and cervical stromal invasion [63]. This new classification resulted in significant stage migration, with many patients being upstaged or downstaged based on the new criteria, like the cases of patients previously classified as Stage I in the 2009 system now classified as Stage II in the 2023 system due to the inclusion of LVSI and molecular markers [64].

The 2009 FIGO staging system for endometrial cancer removed positive peritoneal cytology as a factor that increases the disease stage. This change was made because the importance of positive peritoneal cytology as an independent risk factor was called into question. However, both FIGO and the American Joint Committee on Cancer (AJCC) continue to recommend that peritoneal washings be obtained and the results recorded for prognostic and treatment planning purposes [65]. Despite its exclusion from staging, positive peritoneal cytology has been shown to be an independent prognostic factor associated with poorer outcomes in endometrial cancer, including higher recurrence rates and decreased survival. Therefore, while it does not alter the stage, it remains a significant factor in risk stratification and management decisions [66].

### 4.1. Evolution of FIGO Staging System

The first reports suggesting the importance of sentinel lymph nodes in EC prognosis date back to more than 20 years, and even older sentinel studies are available. Over time, knowledge of EC biology increased, and diagnostic and therapeutic approaches underwent substantial modifications [67]. Despite the old, the widely recognized value of LYNA (Lymph Node Assistant) and the new concept of sentinel lymph node biopsy—endorsed by a large body of the literature on patients, mostly in low-risk cases and employing different methods (radiotracers, blue dye, indocyanine green)—provided evidence of accuracy and pushed the scientific world toward a less invasive therapeutic approach, aiming at producing reductions in the iatrogenic morbidity related to systematic lymphadenectomy [68]. Such changes were not accompanied by proportional modifications of the staging systems. A source of doubt was the fact that this strategic shift toward less invasive treatment was led by the assessment of low-risk cases, thereby reducing the scientific world’s concern regarding modifications of the staging system [69].

Despite refinements, the evolution of the FIGO staging system has mostly depended on further mapping the spread of the disease [70]. In seeking to simplify the discrimination, which originally aimed at choosing the proper treatment, with prognostic information somewhat intrinsic to it, the first FIGO staging system is still the most valuable one in light of the principles of clinical staging [27]. Even though preoperative staging is becoming more and more important because of the treatment strategies shifting more and more in favor of less radical surgical approaches, therapeutic lymphadenectomy remains the main determinant of the prognosis, significantly affecting the regional lymph node status when assessing myometrial invasion [4,36].

### 4.2. Current FIGO Staging Guidelines

Surgical assessment is critical for appropriate staging and important for determining the need for adjuvant therapy. Lymphadenectomy has both a therapeutic and staging role and may be underused in patients presumed to have low-risk disease [62]. Lymph node metastases are an adverse prognostic factor and are associated with an increased risk of recurrence. The surgical specimens should be carefully evaluated postoperatively with attention to the depth of myometrial invasion, involvement of the uterine serosa, and presence/absence of lymphovascular space invasion. Additional pathologic and radiologic investigation may or may not be performed in situations where the intraoperative assessment is questionable [71].

EC is surgically staged according to the FIGO 2009 guidelines. It is clinically staged after an appropriate evaluation of the patient’s history and physical examination, chest X-ray, intravenous excretory urography or CT scan, and preoperative biopsy [72]. Pelvic washing cytology and peritoneal cytology have not been shown to significantly alter the staging. Surgical staging includes a total abdominal or laparoscopic-assisted vaginal hysterectomy, bilateral salpingo-oophorectomy, examination of pelvic and para-aortic lymph nodes (with selective lymphadenectomy acceptable in early lesions), and washing cytology [73].

## 5. Impact of Molecular and FIGO Classification on Diagnosis

The new molecular and FIGO classification takes an important first step in genetically characterizing ECs in order to guide personalized treatment. Incorporating high-throughput techniques, such as next-generation sequencing, into routine clinical care can be challenging and is associated with significant costs and bioinformatic hurdles [57]. At present, because of the rise in incidence and the increase in the number of patients diagnosed with EC, there is a considerable number of patients for whom these new technologies can be applied to provide additional genetic information. Nevertheless, in order for these new modalities to be accessible and useful for all patients with EC, some work needs to be performed to reduce costs and bioinformatic hurdles [59].

EC is one of the few cancers whose incidence is increasing, something that is largely related to the obesity epidemic, making it a cancer of growing public health concern. Over the last several years, our understanding of the molecular basis of EC has greatly improved, even transforming the way we think about the disease at a molecular level, and the new molecular insights have led to a new molecular and translational classification that was first implemented in 2014 by TCGA [20]. In 2021, FIGO published an updated staging and histological classification incorporating the new molecular advances in an effort to better stratify patients at diagnosis, guide adjuvant treatment decisions, and improve outcomes. It is essential for all physicians caring for women with gynecologic cancers to understand these recent molecular and staging changes, as they have a significant impact on the diagnosis, patient counseling, and treatment, regardless of whether the patients are surgical or non-surgical candidates [60].

### Diagnostic Accuracy and Precision Medicine

Several concepts reflecting precision medicine have been drawn in the field of EC. The critical role of the diagnostic biopsy in the assessment of the disease is recognized [56]. As metastatic behavior differs based on different molecules expressed, many algorithms guiding the diagnostic and therapeutic process have included the performance of diverse molecules or gene tests. The molecule test is generally used to determine mismatch repair protein expression or microsatellite instability. The gene tests most commonly indicated are the many panels used for the assessment of the molecular basis of the different ECs [63].

Histological evaluation at biopsy in case of abnormal uterine bleeding has shown to have a high level of diagnostic accuracy, provided an adequate biopsy is performed [43]. Various reports have demonstrated that dilatation and curettage with a low volume of tissue do not represent the architecture of the polyp, myoma, or endometrium with sufficient accuracy and may lead to false negative results [64]. The concept of “diagnostic excision” has, therefore, been developed, which implies that an adequate diagnostic biopsy excises the lesion in toto, disrupting the borders that may obscure the diagnosis if shown in relation [64].

## 6. Therapeutic Advances in EC

EC is one of the most common gynecologic cancers in developed countries. Most patients are diagnosed at an early stage with abnormal uterine bleeding and have a good prognosis with a five-year survival rate [74]. Adjuvant treatment after surgery is generally tailored to the risk of recurrence, including radiotherapy in high-intermediate-risk and pelvic chemoradiotherapy in high-risk postmenopausal women [74]. The role of lymphadenectomy is controversial, not showing a clear therapeutic benefit in the latest studies, but in selected patients for staging purposes [75]. Hormone therapy may be used in early-stage hormone-dependent disease, and chemotherapy may be used in more advanced or aggressive cases [75]. However, some subtypes of EC are more aggressive, not hormone-dependent, and usually found in advanced stages, but they have a worse prognosis with the current available treatments, which often leads to low funding for research on EC and the poor development of new drugs for EC. In fact, no novel agents had been approved for EC over the last ten years, until four new agents developed immunotherapy as a new era for EC [75].

EC is usually an indolent hormone-dependent adenocarcinoma. Once diagnosed in the early stages, the treatment mainly consists of surgery and postoperative hormone therapy or radiotherapy [76]. However, some subtypes of EC are more aggressive, not hormone-dependent, and are usually found in advanced stages, having a worse prognosis with the current available treatments [76]. For this reason, no novel agents had been approved for EC over the last ten years, until four new agents—pembrolizumab, lenvatinib, and dostarlimab—developed immunotherapy as a new era for EC [6,75].

The recommended treatment protocols for POLE ultramutated/POLE-mutated EC are influenced by the tumor’s favorable prognosis and robust immune response. Given the excellent outcomes associated with this subtype, treatment strategies often focus on de-escalation of therapy. The treatment of POLE mutated endometrial carcinoma often involves surgical resection followed by a tailored approach to adjuvant therapy, with a strong consideration for minimizing additional treatments due to the excellent prognosis associated with this molecular subtype (Table 3).

POLE mutations in endometrial carcinoma have a significant impact on treatment resistance, particularly in the context of platinum-based chemotherapy. Studies have shown that POLE-mutated tumors exhibit increased resistance to platinum-based chemotherapy in vitro [69]. This resistance is likely due to the high mutational burden and the resultant robust immune response, which includes increased infiltration of CD4+ and CD8+ T lymphocytes and overexpression of PD-1 on tumor-infiltrating lymphocytes [72]. Despite this resistance to chemotherapy, POLE-mutated endometrial carcinomas are associated with a favorable prognosis. This is attributed to the high immunogenicity of these tumors, which leads to a strong antitumor immune response [78]. The excellent clinical outcomes observed in patients with POLE-mutated tumors suggest that the favorable prognosis is not due to chemotherapy sensitivity but rather to enhanced immunogenicity and immune response [79].

The treatment approach for POLE-mutated EC often involves de-escalation of adjuvant therapy [80]. The American Society for Radiation Oncology (ASTRO) guidelines suggest that radiation therapy alone may be sufficient for patients with POLE ultramutated tumors who are eligible for adjuvant therapy based on clinical and pathologic factors [78]. Additionally, the high mutational burden makes these tumors good candidates for immune checkpoint inhibitors, such as pembrolizumab, particularly in the context of advanced or recurrent disease [80].

The recommended treatments for dMMR EC primarily involve immune checkpoint inhibitors due to the high mutation rates and favorable immune microenvironments associated with these tumors [81]. The NCCN guidelines recommend pembrolizumab for patients with advanced or recurrent dMMR endometrial cancer, particularly after progression on prior therapies. Pembrolizumab has shown significant efficacy in this setting, leading to its FDA approval for dMMR tumors [59]. The KEYNOTE-158 study demonstrated that pembrolizumab has robust and durable antitumor activity in this patient population, with an objective response rate (ORR) of 48% and a median progression-free survival of 13.1 months [81]. Dostarlimab is another anti-PD-1 inhibitor that has demonstrated antitumor activity in dMMR endometrial cancer [82]. The ongoing GARNET phase I trial has shown promising results, with an objective response rate (ORR) of 43.5% in these patients [82].

Combination therapies such as pembrolizumab with lenvatinib have shown efficacy (significantly longer PFS and OS compared to chemotherapy alone) in patients with advanced endometrial cancer, regardless of MSI status, but particularly benefit those with dMMR tumors [72].

Patients with Lynch syndrome-associated endometrial cancer (a subset of dMMR tumors) also exhibit higher response rates to ICIs compared to sporadic dMMR tumors, emphasizing once more the importance of molecular profiling to identify patients who are most likely to benefit from these therapies [83].

The treatment of NSMP EC involves a combination of surgical management, adjuvant therapy, systemic therapy, and participation in clinical trials (Table 4).

The treatment options for CNH/p53 abnormal EC, characterized by frequent copy-number alterations and TP53 mutations, are primarily guided by its aggressive nature and poor prognosis. The NCCN guidelines recommend a multimodality approach, which almost always includes chemotherapy. It generally involves a combination of chemotherapy, immune checkpoint inhibitors, targeted therapies, and potentially radiation therapy, with a strong consideration for clinical trial enrolment to explore emerging treatments. (Table 5).

Personalized Treatment for Endometrial Carcinoma (PETREC) is a prospective Finnish multicenter phase 3 trial which investigates the efficacy of whole pelvic radiotherapy versus vaginal brachytherapy in high-intermediate risk MMRd and NSMP molecular subgroups, and chemotherapy versus chemoradiotherapy in early-stage high-risk p53abn subtype and nonendometrioid carcinomas [85]. Another trial of interest is the phase II study of cabozantinib in recurrent/metastatic endometrial cancer, which includes patients with serous histology, a subtype often associated with CNH. This study has shown some activity in this cohort, supporting further evaluation in genomically characterized patient groups [86].

Neoadjuvant chemotherapy is an upcoming field which might help patients with non-operative candidates’ disease to undergo surgery, and the progress highlights of the study are also presented in the current issue. As always, surgery is the mainstay treatment of EC and, as indicated, is associated with high-tech innovations to offer the best for our patients [87].

Surgery refuses significant changes from recent innovations. Minimally invasive surgery has become the first choice when indicated due to lower intra- and post-operative morbidity. Fertility-sparing surgery, comprising hysteroscopic resection of low-grade EC and progestin therapy of grade 1 EC, is still consolidating in the field, thanks to the published works concerning oncologic outcomes [72]. The lymphadenectomy controversy has been clarified by the recently published papers, and selective lymphadenectomy is indicated to decrease morbidity related to full lymphadenectomy without worsening staging [78].

## 7. Immunotherapy in EC

Although the majority of patients are cured with surgery and adjuvant treatment, there are some high-risk subtypes with poor outcomes, and treatments for advanced, recurrent, or metastatic disease are often not very effective and have a lot of associated toxicity. Recently, there has been a great interest in the field of immunotherapy for many different types of cancers, and a number of immune checkpoint inhibitors have been tested in clinical trials for the treatment of EC with varied results [37].

The tumor microenvironment of EC often has a great number of tumor-infiltrating lymphocytes (TILs), which may be associated with a more favorable prognosis for patients. Given that this is a sign of antitumor immune response in EC, research on immunotherapy is of great interest [78]. Several immune checkpoint inhibitors have already been tested in clinical trials for the treatment of patients with advanced, recurrent, or metastatic EC, and nivolumab and pembrolizumab have been approved by the FDA for some patients. However, at this point, the use of immune checkpoint inhibitors is only approved for a small subset of EC patients, and more research is being performed to test their efficacy in combination with other treatments and to better select the patients who would benefit the most from this therapy [9].

## 8. Combination Therapies and Clinical Trials

Overall, the future management of advanced and recurrent EC is likely to include some degree of molecular profiling along with the development of immunotherapy and the assessment of new drug combinations based on tumor histology, molecular features, or resistance mechanisms [88].

The treatment of advanced or recurrent EC is a challenge, as the response rates are low with single-agent chemotherapy, hormone therapy, or molecularly targeted agents. Several combinations of chemotherapy, targeted therapy, and immunotherapy are currently being investigated in the hope of improving the outcomes [13]. In this chapter, we review the available evidence for combination therapies and ongoing clinical trials, and provide an overview of the completed or ongoing phase II and III trials for systemic therapy in advanced or recurrent EC [6].

## 9. Challenges and Future Directions

One of the most significant challenges is the cost associated with molecular testing. The financial burden of POLE testing and other molecular diagnostics can be substantial, potentially limiting access for many patients. The high cost of these tests may slow their widespread adoption, particularly in resource-limited settings. While specific pricing for POLE assessments may vary by institution and by the method used, it is generally acknowledged that molecular assays incur additional costs compared to histopathological and immunohistochemical evaluations. Surrogate markers, such as immunohistochemical evaluation of MMR or p53 status, are used to replace direct POLE testing in some cases, but although generally less costly, it may not achieve the same prognostic precision. While there is some evidence that POLE-mutated ECs exhibit a higher number of TILs compared to POLE-wild-type ECs, the accuracy of using TILs alone as a surrogate marker is moderate and will not replace the molecular testing [81]. Addressing these economic barriers will ensure equitable access to precision medicine for all EC patients and, in time, lead to long-term cost savings, primarily due to a reduction in overtreatment and prevention of recurrences, which can ultimately lower the overall healthcare expenditure.

Although many studies have tried to clarify the molecular pathogenesis of EC and identify suitable molecular targets, our comprehensive review suggests that a very limited number of molecular-targeted agents have been established. These targeted agents are also not widely used in EC patients, except for those who belong to TCGA subtypes. It is a challenging issue to assess the heterogeneity of EC and determine which patients should be selected for the administration of these agents [12]. Furthermore, the molecular pathogenesis of EC is still not fully understood. It is urgent to expand basic studies and conduct large-scale clinical trials. In addition to genetic changes, the roles of epigenetic changes and the immune environment should also be characterized. Moreover, certain inherited states related to Lynch syndrome and PTEN hamartoma tumor syndrome are known to be associated with an increased risk of EC. The combination of molecular features and inherited states should be highlighted, as it might support the implementation of tailored therapeutic algorithms [17].

## 10. Conclusions

The cornerstone of therapy is still surgical staging, which individualizes the adjuvant treatment. Since there are no randomized trials comparing the different surgical approaches, treatment consists of radical hysterectomy or simple/modified radical hysterectomy for women diagnosed with early disease who are fit for surgery. Lymphadenectomy should also always be performed. In cases of advanced disease, the role of surgery is debulking and could also include pelvic and para-aortic lymph node dissection. Although there is no level I data supporting the lymphadenectomy benefit, several retrospective studies assume therapeutic/prognostic benefit when the disease is staged. To avoid undertreatment or overtreatment, the multidisciplinary team should always discuss the surgical plan.

EC is the seventh in the ranking of the most common cancers in women. The etiology remains the same: estrogen unopposed by progesterone, obesity, and diabetes. It was traditionally divided into two types: type 1, with better prognosis, diagnosed in pre- and perimenopausal women, and associated with estrogen excess; and type 2, diagnosed in postmenopausal women, often non-obese, with a worse prognosis. This classification was performed before molecular biology studies, and currently, the most accepted classification is the one presented by The Cancer Genome Atlas research network, which divides EC into four subtypes: POLE ultramutated, microsatellite instable hypermutated, copy-number low/p53-wild type, and copy-number high. This classification is extremely important because each group has different genetic and molecular mutations, behavior, and prognosis. This information could also be used in the future to guide treatment decisions.

## Figures and Tables

**Table 2:** Molecular Classification and Diagnosis of Endometrial Cancer—a comparative table of key molecular testing recommendations.

Guideline/Society	Universal MMR Testing?	POLE Mutation Testing	p53 IHC Testing	Additional Notes
**WHO (2020)**	Recommended for most ECs	Recommended in certain cases, particularly high-grade	Recommended in high-grade or ambiguous histology	Emphasizes routine molecular classification for improved prognostication and potential targeted therapy.
**ESGO/** **ESTRO/ESP (2021)**	Universal MMR IHC	Recommended in selected subgroups (e.g., high-grade, high-intermediate-risk)	Recommended to identify p53-abnormal phenotype	Integrates molecular subtyping into clinical decision-making; especially important for adjuvant therapy decisions.
**ESMO**	Universal or near universal MMR testing	Encouraged in high-grade tumors for prognostic assessment	Encouraged for further subclassification	Sees molecular classification as essential for accurate risk stratification and therapy guidance.
**NCCN**	Universal for Lynch screening	Consider in select scenarios to refine risk	Consider refining prognostic group	Recommends universal MMR testing but leaves POLE/p53 to the discretion of clinicians based on tumor features and resources.
**SGO**	Universal MMR deficiency screening for Lynch	Optional/Research-based adoption is growing	Optional/Research-based but increasingly used	Emphasizes identifying hereditary risk (Lynch). Adoption of full molecular subtyping is encouraged but may vary by center.

**Table 3:** Recommended treatment protocols for POLE ultramutated/POLE—mutated EC [67,77].

Recommended Protocols in POLE Ultramutated/POLE—Mutated EC	
1. **Surgery**	The primary treatment is surgical resection, typically involving a total hysterectomy with bilateral salpingo-oophorectomy.
2. **Adjuvant Therapy**—due to the favorable prognosis, adjuvant therapy can often be minimized	**Radiation Therapy (RT)**—The American Society for Radiation Oncology (ASTRO) guidelines suggest that external beam radiation therapy (EBRT) alone is a reasonable option for patients with POLE-mutated tumors who are eligible for adjuvant therapy **Chemotherapy** is generally not required, and, if used, it is often in combination with RT for high-risk features
3. **Immunotherapy**	The high mutational burden and immune infiltration in POLE mutated tumors make them good candidates for immune checkpoint inhibitors. These tumors respond well to PD-1 inhibitors like pembrolizumab, but this is more relevant in the context of advanced or recurrent disease rather than as a standard adjuvant therapy.
4. **Observation**	For early-stage disease, observation post-surgery without adjuvant therapy is an option (ongoing trials such as PORTEC-4a and TAPER).

**Table 4:** Recommended treatment protocols for CNL./NSMP EC [59,84].

Recommended Protocols in NSMP EC	
1. **Surgery**	The primary treatment is surgical resection, typically involving a total hysterectomy with bilateral salpingo-oophorectomy. Lymphadenectomy or sentinel lymph node biopsy may be performed to assess lymph node involvement.
2. **Adjuvant Therapy**	For early-stage disease with high-risk features, adjuvant RT or chemotherapy may be recommended. The ASTRO guidelines suggest that adjuvant RT can be beneficial in reducing local recurrence.
3. **Systemic Therapy-** for advanced or recurrent disease	Chemotherapy—Multi-agent chemotherapy regimens such as carboplatin and paclitaxel are commonly used. Hormonal Therapy -Hormonal therapy, including progestins, aromatase inhibitors, or selective estrogen receptor modulators (especially in hormone receptor-positive tumors) Targeted Therapy—The combination of pembrolizumab and lenvatinib has received FDA approval for MMRp/MSIs endometrial cancer
4. **Clinical Trials**	Combining immunotherapy with frontline chemotherapy and other targeted agents and hormonal combinations with cyclin-dependent kinase 4/6 inhibitors and letrozole

**Table 5:** Recommended protocols in CNH/p53abn [82,85].

Recommended Protocols in CNH/p53 Abnormal EC	
1. Chemotherapy.	Typically involves platinum-based chemotherapy, such as carboplatin and paclitaxel, the standard regimen for high-grade and serous-like endometrial cancers.
2. Immune Checkpoint Inhibitors for advanced or recurrent cases	Pembrolizumab, often in combination with lenvatinib, is particularly relevant for MMRp/MSIs endometrial cancer, which includes CNH/p53abn subtypes.
3. Targeted Therapy	Trastuzumab is recommended for HER2-positive serous endometrial cancers, which are primarily p53-abnormal.
4. Radiation Therapy	Especially in cases with high-risk features such as deep myometrial invasion or lymphovascular space invasion.
5. Clinical Trials	Given the associated poor prognosis, participation in clinical trials is highly encouraged They may include new ICIs, targeted therapies, or combination regimens.

## Data Availability

The data are contained within the article.
